# Association of Hearing Impairment and 24-Hour Total Movement Activity in a Representative Sample of US Adults

**DOI:** 10.1001/jamanetworkopen.2022.2983

**Published:** 2022-03-18

**Authors:** Pablo Martinez-Amezcua, Erin E. Dooley, Nicholas S. Reed, Danielle Powell, Bjoern Hornikel, Justin S. Golub, Kelley Pettee Gabriel, Priya Palta

**Affiliations:** 1 Division of General Medicine, Department of Medicine, Columbia University Irving Medical Center, New York, New York; 2 Department of Epidemiology, School of Public Health, University of Alabama at Birmingham; 3 Department of Epidemiology, Johns Hopkins Bloomberg School of Public Health, Baltimore, Maryland; 4 Cochlear Center for Hearing and Public Health, Baltimore, Maryland; 5 Department of Health Policy and Management, Johns Hopkins Bloomberg School of Public Health, Baltimore, Maryland; 6 Department of Otolaryngology–Head & Neck Surgery, Columbia University Irving Medical Center, New York, New York; 7 Department of Epidemiology, Mailman School of Public Health, Columbia University Irving Medical Center, New York, New York

## Abstract

**Importance:**

Lower physical activity (PA) levels have been proposed as a mechanism to explain the association between hearing loss and various adverse outcomes of aging. But whether hearing loss is associated with lower PA is poorly understood.

**Objective:**

To evaluate whether hearing loss is associated with lower levels of PA.

**Design, Setting, and Participants:**

This cross-sectional analysis was conducted in a representative sample of US adults (aged 30-69 years) who participated in the 2011-2012 cycle of the National Health and Nutrition Examination Survey (NHANES). Data analysis was conducted from July to September 2021.

**Exposures:**

Hearing was assessed, in dB, by pure-tone audiometry and summarized as the better hearing ear’s pure-tone average (BPTA) of 4 speech frequencies (500, 1000, 2000, and 4000 kHz); a higher BTPA indicates worse hearing. Hearing was also categorized into normal (BPTA ≤25 dB), mild hearing impairment (>25 to 40 dB), and moderate or greater hearing impairment (>40 dB).

**Main Outcomes and Measures:**

Total 24-hour movement activity was measured using wrist accelerometers worn at all times for 7 consecutive days and summarized in monitor-independent movement summary units (MIMS) units (higher MIMS units indicate more movement). The association between BPTA and MIMS units was evaluated using a multivariable-adjusted (demographic characteristics and chronic conditions) piecewise spline regression (knot at 15 dB). Mean differences in MIMS units across hearing categories compared with normal hearing were estimated.

**Results:**

The study included 2490 participants (mean [SE] age, 48.9 [0.3] years; 900 [weighted percentage, 69.3%] White individuals, 1248 [weighted percentage, 50.8%] female participants). Before the knot at 15 dB, we found that each 10-dB higher BPTA was associated with 860.4 (95% CI, 444.8-1276.1) higher MIMS units. Conversely, after the knot, each 10-dB higher BPTA was associated with 458.6 (95% CI, 27.7-889.4) lower MIMS units. The difference in MIMS units across hearing categories was not statistically significant.

**Conclusions and Relevance:**

In this cross-sectional study, in the range of hearing sensitivity of BPTA of 15 dB or greater, worse hearing was associated with lower MIMS units. Lower PA may be a mechanism contributing to the association between hearing impairment and adverse health.

## Key Points

**Question:**

Is hearing loss associated with lower levels of physical activity?

**Findings:**

In this cross-sectional analysis of 2490 US adults aged 30 to 69 years, worse hearing, as defined by pure-tone audiometry, was associated with lower objectively assessed physical activity levels starting at a 15-dB hearing level threshold.

**Meaning:**

These findings suggest that lower levels of physical activity may be one of the mechanisms explaining the adverse associations between hearing loss and several health outcomes, such as cognition, physical functioning, and hospitalizations.

## Teaser

This cross-sectional study evaluates whether hearing loss is associated with lower levels of physical activity.

## Introduction

Being physically active is important for optimal health at all ages. Higher levels of physical activity are associated with a lower cardiovascular disease risk, increased longevity, and higher quality of life.^1,2,3^ Nevertheless, less than 20% of adults meet the recommended levels of physical activity, which, according to the 2018 US Department of Health and Human Services *Physical Activity Guidelines for Americans*, is at least 150 minutes per week of moderate to vigorous intensity physical activity.^3^ Furthermore, activity levels typically decline with age.^4,5,6,7^

Hearing loss is common and frequently undetected among middle-age and older adults, affecting approximately one-fifth of middle-aged adults and nearly two-thirds of adults older than 65 years.^8,9^ Previous studies suggest that hearing loss may lead to adverse health outcomes, including faster decline in cognitive function, slower gait speed, and poorer balance at older ages.^10,11,12,13^ One mechanism that may explain these associations is lower levels of physical activity among those with impaired hearing.

Previous research has shown that adults with hearing loss perform less physical activity, objectively measured, than those without hearing loss.^14,15,16^ While these previous studies have used accelerometry measures to derive metrics of physical activity, only 1 study^17^ has used physical activity metrics that go beyond time spent at different intensities (ie, sedentary, light, moderate, and vigorous physical activity). Moreover, accelerometry-derived metrics typically are device specific, making it difficult to contrast findings from studies that use different accelerometer devices. Hence, novel physical activity metrics have been developed leveraging accelerometry-collected data that are not specific, such as Monitor-Independent Movement Summary (MIMS) units.^18^ This study aimed to investigate the association between hearing loss measured by pure-tone audiometry and physical activity summarized using MIMS units among the nationally representative National Health and Nutrition Examination Survey (NHANES) sample of adult participants of the 2011-2012 cycle. We hypothesized that participants with higher hearing thresholds in the audiometry test (ie, worse hearing) would have lower levels of physical activity.

## Methods

### Study Population

Our study population included 3012 participants, aged 30 to 69 years, recruited to the 2011-2012 NHANES cycle who completed the audiometric assessment; this NHANES cycle was selected because, of the recent cycles when wrist accelerometry data were collected, pure-tone audiometry was obtained only in 2011-2012. NHANES uses a complex, multistage probability design to oversample racial and ethnic minority groups and individuals with lower income and obtain a nationally representative group of non-institutionalized US residents.^19^ NHANES participants completed an in-person home interview to provide demographic, socioeconomic, and health-related information. Physical examinations were conducted in a mobile examination center. During the examination, participants were invited to wear a physical activity monitor and complete a pure-tone audiometric assessment. We excluded 63 participants who reported hearing aid use because hearing aid use may modify the association between hearing loss and physical activity. We also excluded 444 participants who did not have physical activity data, and 15 participants who had missing information on the covariates of interest. The final analytic sample included 2490 adults aged 30 to 69 years who completed the hearing and physical activity assessments.

The protocol for NHANES was approved by the National Center for Health Statistics institutional review board, and all participants provided written informed consent. Per the Common Rule, institutional review board approval for this analysis was exempt, as NHANES data are deidentified and publicly available. This study followed the Strengthening the Reporting of Observational Studies in Epidemiology (STROBE) reporting guidelines for cross-sectional studies.^20^

### Assessment of Physical Activity in MIMS Units

Movement was measured using the ActiGraph GT3X+ (ActiGraph). NHANES participants were asked to wear the device on the nondominant wrist for 7 consecutive complete days (examination day [day 1] and requested return date [day 9] were not considered), including during water-based activities (eg, bathing and swimming). The ActiGraph contains a triaxial accelerometer that was programmed to collect data at 80 Hz.^21^ Raw acceleration data were downloaded and summarized into MIMS units at 1-minute bouts using a nonproprietary algorithm.^18^ The sum of the 1440 minute-level MIMS units collected each day represents the total amount of movement activity throughout 24 hours. For our analyses, we summed the triaxial MIMS units across all valid days and then calculated the mean.

To reduce measurement error, as is commonly done in research using data from wrist-worn accelerometers,^22^ and in adherence with the NHANES accelerometry protocol,^23^ days in which wear time was incomplete (<1440 minutes) or with 17 or more hours of sleep (determined by a previously established algorithm) were classified as nonvalid and excluded. All participants with at least 1 day of valid wear were included in our analyses since previous studies have determined that 1 day is sufficient for population-level analyses.^24^

### Assessment of Hearing

Hearing was assessed by trained NHANES staff using pure-tone audiometry. The tests were performed in a sound-attenuated booth with a calibrated audiometer in the mobile examination center. Air conduction hearing thresholds were identified using best-practice threshold seeking methods^25^ in dB hearing level (HL) at different frequencies for each ear separately. We calculated a 4-frequency (0.5, 1.0, 2.0, and 4.0 kHz) pure-tone average for each ear and used the better-hearing ear’s pure-tone average (BPTA) as a continuous measure for analysis. Consistent with previous literature, we further categorized participants hearing status into normal hearing (BPTA ≤25 dB HL), mild hearing loss (BPTA >25 to 40 dB HL), and moderate or greater hearing loss (BPTA >40 dB HL).^9,26^

### Assessment of Covariates of Interest

Our demographic covariates of interest included age, sex, race and ethnicity (Mexican American, other Hispanic, non-Hispanic White, non-Hispanic Black, non-Hispanic Asian, and other races [American Indian or Alaska Native, Native Hawaiian or Pacific Islander, multiple races or ethnicities, or unknown]), education (<high school, high school completed, and >high school), marital status (married vs not) and employment status (currently working vs not). We also included some anthropometric measures and chronic conditions, such as body mass index (BMI; calculated as weight in kilograms divided by height in meters squared), diabetes (ever been told by a doctor you have diabetes, fasting glucose ≥126 mg/dL [to convert to millimoles per liter, multiply by 0.0555], or hemoglobin A_1c_ [HbA_1c_] ≥6.5% [to convert to proportion of total hemoglobin, multiply by 0.01]), hypertension (ever been told by a doctor that you have high blood pressure, measured systolic blood pressure ≥140 mm Hg, or measured diastolic blood pressure ≥90 mm Hg), smoking status (current, ever, never), heart attack (ever been told by a doctor you had a heart attack), stroke (ever been told by a doctor you had a stroke), and heart failure (ever been told by a doctor you had heart failure). We selected this set of covariates for adjustment in our analysis based on their documented associations with both hearing and physical activity, making them potential confounders of our association of interest.

### Statistical Analysis

In descriptive analyses, to compare demographic and medical characteristics across the hearing loss groups, we used analysis of variance and χ^2^ tests for continuous and categorical variables, respectively. To estimate the association between hearing loss and MIMS units, we used multivariable-adjusted regression models that accounted for the NHANES complex sampling design (sampling weights). We built 2 models: model 1, adjusted for age, sex, race and ethnicity, education, marital status, employment status, BMI, diabetes, hypertension, and smoking status. Model 2 additionally adjusted for stroke, heart attack, and heart failure.

Initial exploratory data analysis, via 2-way plots with locally weighted smoothing curves, revealed a nonlinear association between MIMS units and BPTA; thus, we introduced a 2-piece linear spline term for BPTA with the knot at 15 dB HL, the value at which we observed a change in the curve. We assessed the mean differences in MIMS units per 10-dB HL higher BPTA (worse hearing) using 2-piece linear spline regression models. We also estimated the mean difference in MIMS units across hearing loss groups (normal, mild, and moderate or greater).

In sensitivity analyses, we examined changing the knot in our spline regression to 20 dB HL (rather than 15 dB HL) to match the current World Health Organization (WHO) thresholds for distinguishing normal hearing from mild hearing loss.^27^ We also examined a knot at 25 dB HL to match former WHO guidelines and the previous literature.^8,9^ Moreover, we created a model with restricted cubic spline terms that model this association more flexibly. Additionally, we created 4 activity groups using age- and sex-specific quartiles of MIMS units previously developed using NHANES data,^21^ and used ordinal logistic regression models to evaluate whether participants with hearing loss had a higher odds of being in a lower activity group. All analyses were conducted using Stata version 15 (StataCorp), and the svy commands were used to account for the complex sampling design of NHANES. We considered *P* < .05 statistically significant, and all tests were 2-tailed.

## Results

Among the 2490 participants, 191 (7.7%) had mild, moderate, or severe hearing loss. The age range was 30 to 69 years (mean [SE], 48.9 [0.3] years), 1248 (weighted percentage, 50.8%) were female; 900 (weighted percentage, 69.3%), White; 695 (weighted percentage, 11.1%), Non-Hispanic Black; and 267 (weighted percentage, 7.2%), Mexican American. In weighted, unadjusted comparisons (Table 1), participants with hearing loss were older; more often male, White, and of lower educational status; more likely to have hypertension and a history of smoking; and less likely to be employed at the time of the examination.

**Table 1.** Weighted Contrast of the Study Population’s Characteristics Across Hearing Groups^a^

Characteristic	Participants, No. (weighted %)				*P* value
	Total (N = 2490)	BPTA ≤25 dB HL (n = 2299)	BPTA >25-40 dB HL (n = 163)	BPTA >40 dB HL (n = 28)	
Age, mean (SE)	48.9 (0.3)	48.2 (0.3)	57.5 (0.9)	58.0 (1.7)	<.001
Sex					
Female	1248 (50.8)	1179 52.1	58 (36.0)	6 (30.2)	.008
Male	1242 (49.2)	1120 (47.9)	103 (64.0)	22 (69.8)	
Race and ethnicity					
Mexican American	267 (7.2)	244 (7.4)	20 (4.9)	3 (6.0)	.06
Non-Hispanic					
Black	695 (11.1)	653 (11.5)	36 (6.2)	6 (9.2)	
White	900 (69.3)	816 (68.4)	71 (79.8)	13 (74.1)	
Other Hispanic	256 (5.6)	234 (5.7)	19 (3.7)	3 (5.5)	
Other^b^	372 (6.8)	352 (6.9)	17 (5.5)	3 (5.3)	
Education					.01
<High school	531 (14.9)	458 (14.1)	61 (24.8)	12 (29.0)	
High school	531 (20.2)	486 (19.9)	486 (23.4)	38 (15.6)	
>High school	1428 (64.9)	1355 (66.0)	64 (51.8)	9 (55.4)	
Married	1129 (39.6)	1043 (39.4)	71 (42.5)	15 (39.1)	.63
Currently working	1557 (69.2)	1467 (69.9)	85 (64.0)	5 (27.4)	.02
BMI, mean (SE)	29.3 (0.2)	29.3 (0.2)	28.9 (0.8)	29.0 (0.9)	.80
Smoking status					
Never	1377 (53.4)	1295 (54.4)	68 (41.6)	14 (49.4)	.08
Former	562 (24.8)	507 (24.8)	47 (26.2)	48 (19.5)	
Current	551 (21.7)	497 (20.9)	48 (32.2)	6 (31.1)	
Diabetes	424 (12.2)	377 (11.8)	40 (16.8)	7 (11.9)	.21
Hypertension	1068 (38.5)	955 (37.5)	97 (50.4)	16 (53.7)	.03
Stroke	69 (1.7)	57 (1.6)	10 (3.6)	2 (5.2)	.05
Heart attack	59 (2.3)	49 (2.2)	10 (2.6)	0 (5.5)	.61
Heart failure	62 (2.0)	54 (1.9)	8 (2.8)	0 (5.5)	.44

Abbreviations: BMI, body mass index (calculated as weight in kilograms divided by height in meters squared); BPTA, better-hearing ear pure tone average; HL, hearing level.

^a^ Accounting for sampling weights.

^b^ Other race includes American Indian or Alaska Native, Native Hawaiian or Pacific Islander, multiple races or ethnicities, or unknown.

In our 2-piece linear spline adjusted regression analysis (Figure), we observed a change in the direction of the association between hearing and movement activity before and after the knot at 15 dB HL; before the knot at 15 dB HL of BPTA, each 10-dB HL higher BPTA was associated with 860.4 (95% CI, 444.8 to 1276.1) higher MIMS units. Conversely, after the knot at 15 dB HL of BPTA, each 10-dB HL higher BPTA was associated with −458.6 (95% CI, −889.4 to −27.7) lower MIMS units.

**Figure.**
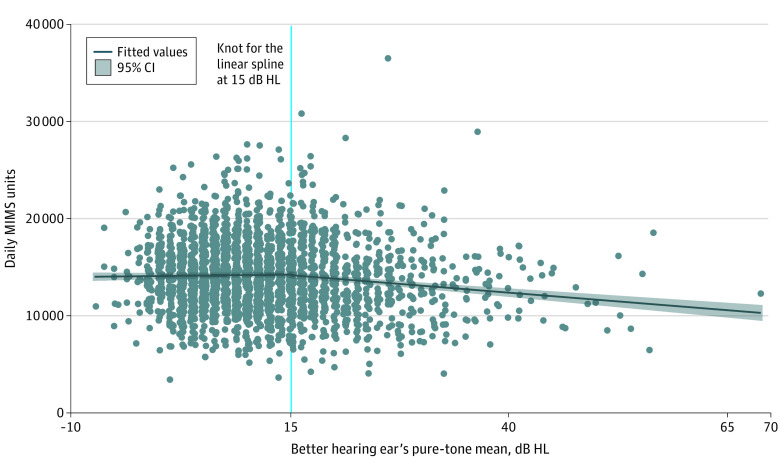
Association Between Better Ear’s Pure-Tone Average and Daily Monitor Independent Movement Summary (MIMS) Units Adjusted for covariates in model 2, ie, age, sex, race and ethnicity, education, marital status, employment status, body mass index, diabetes, hypertension, smoking status, stroke, heart attack, and heart failure. β coefficients were estimated with multivariable adjusted linear regression. Higher MIMS units indicates more movement. HL indicates hearing level.

The mean differences in MIMS units across hearing loss groups are provided in Table 2. Compared with participants with normal hearing, participants with mild hearing loss had, a mean of −54.1 (95% CI, −896.9 to 788.8) fewer MIMS units, and those with moderate or greater hearing loss had a mean of −1278.6 (95% CI, −3216.5 to 659.4) fewer MIMS units, but these differences were not statistically significant.

**Table 2.** Association Between Hearing Loss Categories and Physical Activity

Hearing level	Mean difference in daily MIMS units, β coefficient (95% CI)^a^	
	Model 1	Model 2
Normal hearing; BPTA <25 dB HL	[Reference]	[Reference]
Mild hearing loss; BPTA >25-40 dB HL	−59.3 (−903.1 to 784.6)	−54.1 (−896.9 to 788.8)
Moderate or greater hearing loss; BPTA >40 dB HL	−1307.2 (−3244.3 to 629.8)	−1278.6 (−3216.5 to 659.4)

Abbreviations: BPTA, better hearing ear’s pure-tone average; HL, hearing level; MIMS, monitor-independent movement summary.

^a^ Coefficients estimated with multivariable adjusted linear regression models using the svy commands in Stata to account for sampling weights. Model 1 was adjusted for age, sex, race and ethnicity, education, marital status, employment status, body mass index, diabetes, hypertension, smoking status. Model 2 was adjusted for covariates in model 1 and stroke, heart attack, and heart failure.

### Sensitivity Analyses

In changing the knot to 20 or 25 dB HL (eFigure in the Supplement), to match the current WHO threshold for mild hearing loss (20 dB HL) as well as the former WHO threshold and most of the previous studies in the field (25 dB HL), we observed the same pattern of associations as with the knot at 15 dB HL: a positive association between BPTA and MIMS units before the knot, and an inverse, although not statistically significant, association after the knot (estimates shown in the eFigure in the Supplement). The model that used restricted cubic spline terms showed a similar pattern (eFigure in the Supplement): individuals with scores in the lower end of the BPTA range had more physical activity, and those with scores in the higher end of the BPTA range had less physical activity. The coefficients for the cubic terms were not statistically significant.

In adjusted ordinal logistic regression models, each 10-dB HL higher BPTA before the 15 dB knot was associated with 27% lower odds of being in a lower physical activity group (eg, in the third vs fourth quartile: odds ratio [OR], 0.73; 95% CI, 0.57-0.94). After 15 dB HL knot, however, each 10-dB HL higher BPTA had higher odds of being in a lower physical activity group, but this finding was not statistically significant (OR, 1.23; 95% CI, 0.98-1.55). Similarly, mild and moderate or greater hearing loss had non–statistically significantly higher odds of being in a lower activity group (mild hearing loss: OR, 1.08; 95% CI, 0.70-1.67; moderate or greater hearing loss: OR, 1.41; 95% CI, 0.58-3.44).

## Discussion

In a weighted analysis representative of the US population aged 30 to 69 years, higher BPTA was associated with lower MIMS units. Our findings from secondary analyses that compared differences in mean MIMS units and odds of being in lower activity categories across hearing loss categories supported our hypothesis that participants with poorer hearing had lower levels of physical activity. While these results were not statistically significant, it is likely that this is due to a relatively low number of participants in the mild and moderate or greater hearing loss groups.

Our findings agree with those of Kuo et al,^17^ who recently evaluated this association using data from participants aged 60 to 69 years from the 2003-2004 NHANES cycle. While both analyses arrived at similar conclusions, our sample included a wider age range and used novel activity metrics that are monitor independent. We also accounted for marital status and employment status, which were not considered in previous papers, but may confound the association between hearing loss and physical activity. Moreover, our findings of a positive association between hearing and physical activity in the range of normal hearing (BPTA <20 dB HL) but a negative association in the range of mild to more severe loss (BPTA ≥20 dB HL) is novel. One of the mechanisms that may explain the association between hearing loss and lower physical activity is that hearing loss may lead to social isolation^28,29^ and reduced life space mobility,^30^ which limit an individual’s opportunities to be physically active.^31^ This mechanism, however, would only be true for those adults for whom the degree of hearing loss is high enough to affect their social life, which is supported by our findings from the linear spline regressions. Another mechanism that may explain the associations we observed is the reduced perception of environmental cues that help with spatial calibration when moving among those with hearing loss.^32^ The nonlinearity that we observed in the association between hearing loss and daily movement has also been described for the association with depressive symptoms and cognition.^33,34^ While the previous literature in the hearing loss field has used the 25-dB HL threshold to define mild hearing loss, there is a lack of consensus on what BPTA should be used to define hearing loss. In the most recent world report on hearing from the WHO, a threshold of 20 dB HL is used.^27^ Moreover, even lower thresholds have been proposed, such as 15 dB HL.^35^

### Limitations and Strengths

Our study has some limitations. First, this is a cross-sectional analysis; thus, temporality cannot be ascertained. Second, during the 2011-2012 NHANES cycle, adults older than 69 years were excluded from audiometric testing; therefore, we may not be observing many NHANES participants in whom hearing loss may be more prevalent and severe. Additional studies confirming these associations in older populations are warranted. Third, there are currently no minute-level MIMS units thresholds to define physical activity intensities.^21^ Therefore, our findings cannot be related to the physical activity recommendations for Americans (≥150 minutes/week of moderate activity or ≥75 minutes/week of vigorous activity), which are widely used for public health messaging. Fourth, our sample size for the group with moderate or greater hearing loss was small (n = 28), which may have limited our statistical power to detect differences in physical activity levels across hearing loss groups. Finally, given the small number of participants who reported hearing aid use, we decided to exclude them from our analysis; thus, we were unable to assess whether hearing aid users were more active than nonusers.

Despite these limitations, our study has several strengths. As additional devices to objectively measure physical activity are implemented in research studies, a comparison of results across studies becomes more challenging since many studies have used device-specific algorithms to summarize accelerometry data. Our study incorporated the use of MIMS units, which use a nonproprietary open-source and device-independent algorithm to objectively derive movement from raw accelerometry data. Thus, MIMS units may help with harmonization efforts across studies. We conducted a series of sensitivity analyses, including moving the knot of our linear spline to evaluate different thresholds at which we observed the association between hearing and physical activity. All analyses suggest that adults with hearing loss were less active; however, only the analysis with a linear spline were statistically significant.

## Conclusions

Our study supports the hypothesis that hearing loss is associated with lower levels of physical activity, characterized using novel MIMS units metrics. Reductions in physical activity as a result of hearing loss may be an important mechanism contributing to the association between hearing loss and adverse health outcomes but will require further investigation in longitudinal studies. Furthermore, whether hearing rehabilitation strategies may help attenuate the adverse consequences of hearing loss on physical activity and other downstream outcomes requires examination in future studies.^36,37^

## Notes

Supplement. **eFigure.** Sensitivity Analyses: Linear Spline Terms With Knots at Different Positions and Cubic Terms
