# Supplementary material for: Association of Hearing Impairment and 24-Hour Total Movement Activity in a Representative Sample of US Adults
Source: JAMA Netw Open. 2022 Mar 18;5(3):e222983. doi: 10.1001/jamanetworkopen.2022.2983 (PMC8933734; doi:10.1001/jamanetworkopen.2022.2983)
Supplement: Supplement. — eFigure. Sensitivity Analyses: Linear Spline Terms With Knots at Different Positions and Cubic Terms [file jamanetwopen-e222983-s001.pdf]

Supplemental Online Content

Martinez-Amezcua P, Dooley EE, Reed NS, et al. Association of hearing impairment and 24-hour total movement activity in a representative sample of US adults. *JAMA Netw Open.* 2022;5(3):e222983. doi:10.1001/jamanetworkopen.2022.2983

eFigure. Sensitivity Analyses: Linear Spline Terms With Knots at Different Positions and Cubic Terms

This supplemental material has been provided by the authors to give readers additional information about their work.

© 2022 Martinez-Amezcua P et al. *JAMA Network Open.*

eFigure. Sensitivity Analyses: Linear Spline Terms With Knots at Different Positions and Cubic Terms

A. Knot at 20 dB HL

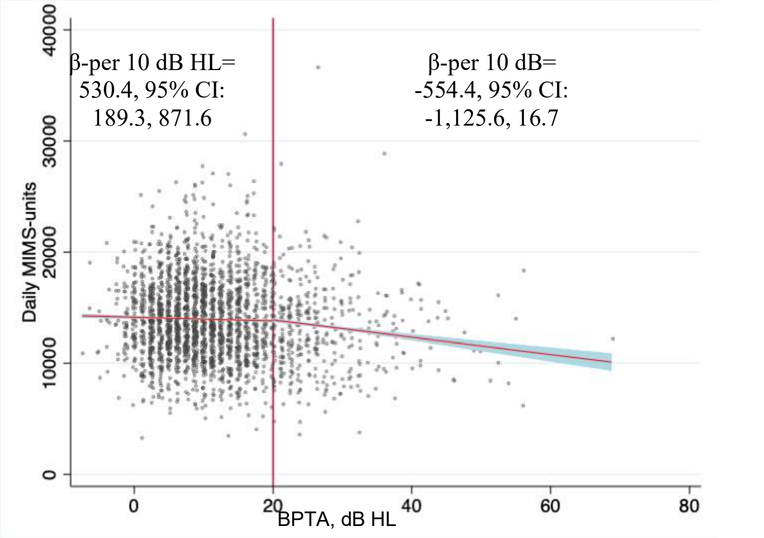

Adjusted for covariates in model 2: age, sex, race/ethnicity, education, marital status, BMI, diabetes, hypertension, smoking status, and stroke, heart attack, and heart failure.
β-coefficients estimated with multivariable-adjusted linear regression adding a linear spline with the knot at 20 dB HL
Abbreviations: MIMS-units=Monitor-independent movement summary units (higher MIMS-units= more movement); dB HL= decibels hearing level

B. Knot at 25 dB HL

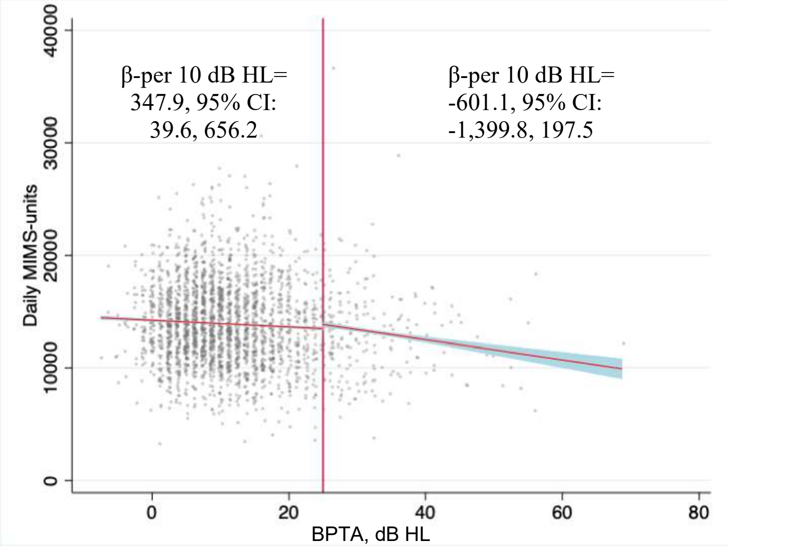

Adjusted for covariates in model 2: age, sex, race/ethnicity, education, marital status, BMI, diabetes, hypertension, smoking status, and stroke, heart attack, and heart failure.
β-coefficients estimated with multivariable-adjusted linear regression adding a linear spline with the knot at 25 dB HL
Abbreviations: MIMS-units=Monitor-independent movement summary units (higher MIMS-units= more movement); dB HL= decibels hearing level

C. Cubic terms, 5 knots

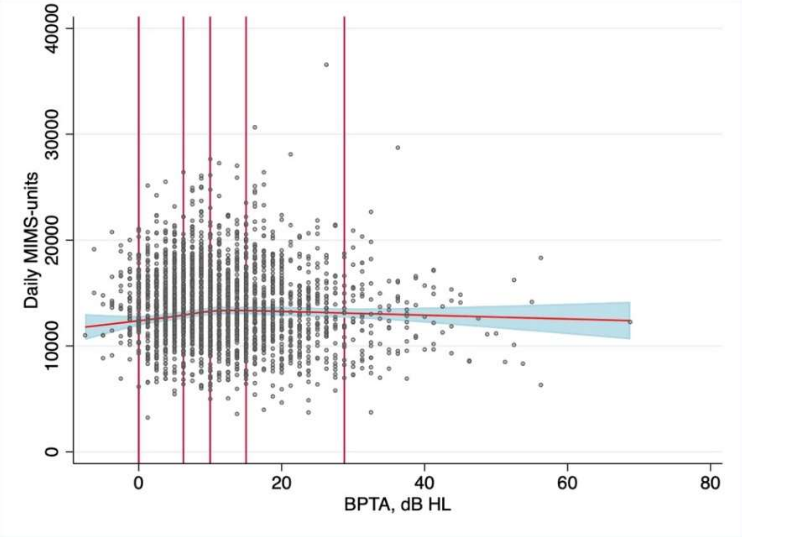

Adjusted for covariates in model 2: age, sex, race/ethnicity, education, marital status, BMI, diabetes, hypertension, smoking status, and stroke, heart attack, and heart failure. β-coefficients estimated with multivariable-adjusted linear regression adding splines with cubic terms with 5 knots, the positions were determined by Harrell's default percentiles.
Abbreviations: MIMS-units=Monitor-independent movement summary units (higher MIMS-units= more movement); dB HL= decibels hearing level

© 2022 Martinez-Amezcua P et al. *JAMA Network Open.*
